# Tuina combined with pulsed dry cupping for constipation and gastrointestinal recovery in severe traumatic brain injury: a multidimensional rehabilitation study

**DOI:** 10.3389/fneur.2026.1828783

**Published:** 2026-08-25

**Authors:** Wenjuan Huang, Hongyi Sun, Yuexiang Zhao, Yuxia Pan

**Affiliations:** 1 Department of Neurosurgery, Taizhou Affiliated Hospital of Nanjing University of Chinese Medicine, Taizhou, Jiangsu, China; 2 Department of Nursing, The Affiliated Taizhou Hospital of Nanjing University of Chinese Medicine, Taizhou, Jiangsu, China; 3 Intensive Care Unit, The Affiliated Taizhou Hospital of Nanjing University of Chinese Medicine, Taizhou, Jiangsu, China

**Keywords:** constipation, gastrointestinal function, pulsed dry cupping, severe traumatic brain injury, traditional Chinese medicine rehabilitation nursing, Tuina

## Abstract

**Objective:**

To evaluate the efficacy of Tuina combined with pulsed dry cupping for constipation management and gastrointestinal (GI) function recovery in severe traumatic brain injury (sTBI), and its associations with nutritional status, neurological function, and patient satisfaction.

**Methods:**

This study was a prospective real-world observational cohort study. A total of 120 patients with severe traumatic brain injury (sTBI) were consecutively enrolled between March 2023 and March 2024. Patients were classified into routine care or combined intervention groups according to routine clinical care pathways without randomization or investigator-driven assignment. Participants in the combined intervention group received Tuina combined with pulsed dry cupping once daily, five times per week for 4 consecutive weeks. Outcomes included constipation-related measures, gastrointestinal function, nutritional indicators, neurological function, and patient-reported outcomes. Appropriate statistical methods were applied for comparisons.

**Results:**

Post-intervention, the combined intervention group showed increased completely spontaneous bowel movements, reduced defecation effort, higher BSS scores, and lower PAC-SYM and GSRS scores versus the routine care group (*p* < 0.05). Serum Hb, ALB, and PA levels and GCS scores were higher in the combined intervention group (*p* < 0.05), while GFAP and NSE levels showed non-significant decreasing trends. PGIC scores were lower in the combined intervention group (*p* < 0.05).

**Conclusion:**

In this prospective real-world observational cohort study, Tuina combined with pulsed dry cupping was associated with improvements in constipation-related symptoms, gastrointestinal function, nutritional indicators, and patient-reported outcomes in patients with severe traumatic brain injury. However, given the observational design, causal inferences regarding efficacy cannot be established.

## 1 Introduction

Severe traumatic brain injury (sTBI) represents a critical neurological emergency with high mortality and disability rates. Early management and multisystem supportive care critically influence patient outcomes. Due to factors such as impaired consciousness, prolonged immobilization, physiological stress responses, and extended use of sedative medications, sTBI patients are highly susceptible to gastrointestinal (GI) dysfunction, particularly constipation. According to relevant studies, the incidence of constipation among hospitalized sTBI patients may exceed 60%. Beyond compromising nutritional intake and delaying rehabilitation, constipation can lead to abdominal distension, aspiration, infections, and even fluctuations in intracranial pressure, thereby elevating the risk of severe complications (1, 2).

The pathogenesis of constipation in this population is complex and multifactorial, involving reduced intestinal peristalsis, autonomic nervous system dysregulation, medication effects, and alterations in daily living routines (3–5). For sTBI patients, the restoration of GI function is not only a fundamental objective of nursing care but also a pivotal determinant of overall rehabilitation progress. However, conventional management strategies, such as oral laxatives and enemas, frequently offer only transient symptomatic relief and are associated with challenges including drug tolerance, adverse effects, and patient dependence (6). Consequently, there is an urgent need to explore safe, effective, and sustainable non-pharmacological interventions for constipation. Recent evidence has further emphasized that gastrointestinal dysfunction in patients with severe brain injury is closely related to brain–gut axis dysregulation and neurocritical care-related factors. Disruption of central autonomic regulation, together with systemic inflammatory responses and prolonged intensive care interventions, may collectively contribute to impaired gastrointestinal motility and constipation in this population (7, 8).

In recent years, the application of non-pharmacological interventions grounded in rehabilitation medicine principles for managing functional impairments in critically ill patients has garnered increasing attention (9). Tuina, a manual therapy based on mechanical stimulation, applies rhythmic abdominal compression and stretching to activate visceral afferent pathways, thereby promoting intestinal peristalsis and enhancing defecatory propulsion. Pulsed dry cupping, characterized by cyclical negative pressure stimulation combined with localized thermal effects, may synergistically enhance mechanical stimulation of the abdominal viscera, improve regional microcirculation, and modulate autonomic nervous system activity (10). The combined use of these two modalities holds promise for more effectively facilitating GI function recovery without increasing pharmacological burden.

From a holistic rehabilitation perspective, alleviating constipation not only restores normal defecation but may also enhance tolerance to enteral nutrition (EN), improve nutritional absorption, and attenuate systemic stress responses. These improvements may, in turn, create more favorable conditions for neurological recovery and overall patient rehabilitation. Despite this potential, research investigating the application of Tuina combined with pulsed dry cupping specifically for constipation management and GI function recovery in sTBI patients remains limited. Moreover, existing studies often focus on single outcome measures, lacking a systematic, multidimensional evaluation of comprehensive rehabilitation effects.

Accordingly, this study adopted a multidimensional rehabilitation research framework to investigate the efficacy of Tuina combined with pulsed dry cupping in managing constipation and promoting GI function recovery in sTBI patients. By comprehensively assessing constipation symptoms, GI function, nutritional status, neurological function-related indicators, and patient-reported perceptions, this study aims to provide evidence-based support for the integration of non-pharmacological rehabilitation interventions into neurocritical care and rehabilitation management.

## 2 Materials and methods

### 2.1 Study participants

This investigation employed a prospective real-world observational cohort study design. Patients diagnosed with sTBI and consecutively admitted to the Neuroscience Intensive Care Unit (ICU) and Neurosurgical ICU of our hospital between March 2023 and March 2024 were enrolled. The clinical efficacy of Tuina combined with pulsed dry cupping in constipation management and GI function recovery was observed. Based on the actual rehabilitation nursing regimen received during hospitalization, participants were allocated to a combined intervention group or a routine care group. This study adhered to real-world clinical practice principles. Patients were not randomly assigned or prospectively allocated by the investigators. Instead, group classification was based on the actual rehabilitation nursing strategies received during hospitalization as part of routine clinical care pathways. The study protocol was approved by the Ethics Committee of our hospital and complied with the ethical standards outlined in the *Declaration of Helsinki*.

Inclusion criteria were: (1) confirmed diagnosis of sTBI based on imaging findings; (2) development of constipation during hospitalization, defined as the absence of spontaneous bowel movements for ≥ 3 consecutive days; (3) relatively stable vital signs permitting tolerance of rehabilitation nursing interventions; and (4) provision of informed consent by the patient or their legal representative.

Exclusion criteria were: (1) history of severe chronic constipation or functional GI disorders; (2) concurrent acute abdominal conditions such as intestinal obstruction, perforation, or GI hemorrhage; (3) coexisting severe cardiac, hepatic, or renal insufficiency, or malignant neoplasms; (4) presence of abdominal skin lesions, infections, or other conditions contraindicating Tuina or cupping therapy; and (5) inter-hospital transfer, discharge, or incomplete data during the study period.

Although a formal power-based sample size calculation was not performed due to the real-world observational design, a sample size justification was conducted based on clinical feasibility and outcome variability. As a prospective real-world observational cohort study, sample size determination was primarily based on statistical robustness for the primary outcome measures and clinical feasibility. Reference to previous literature and preliminary clinical observations at our center indicated considerable variability in constipation relief indicators among sTBI patients receiving standard care (11). To ensure statistical stability for the analysis of primary endpoints (e.g., frequency of completely spontaneous bowel movements and constipation symptom scores) and to accommodate between-group comparisons and multidimensional analyses, an initial target of at least 50 patients per group was set. Considering potential attrition due to loss to follow-up, transfer, or incomplete data, the final sample size was set at 60 patients per group, yielding a total of 120 participants with equal group classification. This sample size was deemed adequate to meet the requirements of primary outcome comparisons and statistical methods including principal component analysis (PCA).

### 2.2 Interventions

Participants were assigned to either the routine care group or the combined intervention group based on the actual constipation management and rehabilitation nursing protocol they received during hospitalization. Consistent with real-world clinical nursing practice, no artificial group classification of treatment or nursing plans was performed. The intervention and observation period for both groups was 4 weeks, commencing immediately following enrollment and baseline assessment, with outcome evaluation conducted at the end of the 4th week.

Participants in the routine care group received standard hospital constipation management protocols, detailed as follows:

(1) Dietary and nutritional management: When permitted by the patient’s clinical condition, dietary structure was adjusted according to the nutritional support plan, with appropriate augmentation of dietary fiber supply. For patients receiving EN, the type of nutritional formula and infusion rate were adjusted based on GI tolerance.

(2) Fluid management: Provided circulatory stability was not compromised, patients were maintained at an appropriate fluid intake level according to routine clinical practice to meet basic physiological requirements.

(3) Basic abdominal care: Measures to preserve abdominal warmth and standard abdominal nursing care were implemented; no additional targeted manual interventions were performed.

(4) Assisted defecation measures: When standard nursing interventions proved insufficient to effectively ameliorate constipation, assisted defecation procedures were employed under physician guidance, in accordance with standardized ward nursing protocols. These measures included administration of osmotic or stimulant laxatives, use of glycerin enemas, or low-volume enemas when necessary. All such interventions were documented in the nursing records.

Participants in the combined intervention group received the combined Tuina and pulsed dry cupping intervention in addition to the standard constipation management protocols, based on the clinical nursing pathway and individual patient tolerance. All procedures were performed by rehabilitation nursing staff who had undergone standardized training. Vital signs and local skin responses were closely monitored throughout the intervention period. Specific procedures were as follows:

(1) Tuina intervention: Prior to the intervention, patients were instructed to empty their bladders and were positioned supine with the abdomen exposed. The procedure predominantly involved mechanical stimulation of the abdomen, with manual techniques applied gradually from light to firm pressure, maintaining a rhythmic and uniform tempo. The entire procedure lasted approximately 10–15 min. Specific manipulations included: ① Using the palm to perform clockwise and counterclockwise circular kneading and pressing motions centered on the umbilicus, 80 repetitions in each direction; ② Applying sustained thumb pressure to specific corresponding points on both sides of the abdomen, approximately 3 min per site; ③ Using the thenar eminence to perform pushing and kneading motions along the anatomical course of the colon, proceeding from proximal to distal segments for approximately 60 repetitions, aimed at promoting intestinal peristalsis and defecatory propulsion. Tuina was administered 1 h postprandially, with attention to patient comfort. The procedure was immediately terminated upon any significant discomfort.

(2) Pulsed dry cupping intervention: Pulsed dry cupping was performed immediately following the Tuina session. Patients were positioned either supine or prone, depending on the target treatment area. Prior to the procedure, a lubricating medium was applied evenly to the treatment area to minimize skin friction. A pulsed negative pressure device was employed for dry cupping. The intensity and frequency of negative pressure were adjusted according to individual patient tolerance to prevent skin damage. The intervention comprised the following steps: ① Pulsed abdominal stimulation: The abdomen served as the primary treatment area, with cyclical negative pressure stimulation applied in a clockwise direction to enhance mechanical stimulation of abdominal viscera; ② Localized thermal stimulation: The combined effects of negative pressure and localized warmth were utilized to promote regional blood circulation and augment GI responses; ③ Adjunctive stimulation of dorsal regions: When indicated, supplementary stimulation was applied to corresponding areas of the back to potentiate the overall therapeutic effect. A single pulsed dry cupping session lasted approximately 10 min. Treatment was continued until mild, localized skin erythema and satisfactory subjective tolerance reported by the patient.

(3) Intervention frequency and duration: Participants in the combined intervention group received the treatment protocol once daily, 5 times per week, for a total of 4 consecutive weeks. If significant discomfort, abnormal skin reactions, or changes in clinical condition occurred during the intervention, the procedure was promptly adjusted or discontinued.

To improve reproducibility, the intervention procedures were standardized before study initiation. All Tuina and cupping procedures were performed by trained rehabilitation nursing staff following a unified protocol. Treatment adherence was monitored through daily nursing records, including session completion and duration. Routine co-interventions such as laxatives, enemas, nutritional support, and standard nursing care were applied uniformly according to clinical indications and documented throughout the study period.

All interventions and clinical management in this study were conducted in accordance with routine clinical practice in the ward. The research team did not interfere with treatment decisions, indications, or nursing procedures. Patients were observed and classified according to their actual clinical care pathways, reflecting a real-world clinical practice setting.

### 2.3 Outcome measures

Given the impaired consciousness and limited ability for self-report in patients with severe traumatic brain injury, PAC-SYM, GSRS, and PGIC assessments were completed using proxy reporting by trained nursing staff and/or caregivers based on standardized observation and patient response.

#### 2.3.1 Assessment of constipation recovery

Weekly frequency of completely spontaneous bowel movements, degree of defecation effort, Bristol Stool Scale (BSS) score (12), and Patient Assessment of Constipation Symptoms (PAC-SYM) score (13) were collected for both groups before and after the intervention to evaluate constipation symptoms. Specific scoring criteria were as follows: (1) Weekly frequency of completely spontaneous bowel movements: Defined as the number of bowel movements per week occurring naturally without any assistive means (e.g., laxatives, enemas). Scoring: 0 = no complete bowel movement; 1–2 = mild constipation; 3–4 = moderate constipation; ≥ 5 = normal. (2) Degree of defecation effort: Rated based on the level of straining required during defecation, on a scale from 0 (no straining) to 4 (severe straining). (3) BSS: This scale classifies stool into 7 types, each representing a distinct stool form. Types 1–2 indicate constipation, types 3–5 indicate normal stool, and types 6–7 indicate diarrhea. Scores range from 1 to 7 based on stool type. (4) PAC-SYM: This scale comprises three subscales: stool symptoms, rectal symptoms, and abdominal symptoms. Each item is scored from 0 to 4, with 0 indicating absence of symptoms and 4 indicating very severe symptoms. Total score ranges from 0 to 48.

#### 2.3.2 Assessment of GI function

GI function status before and after the intervention was evaluated using the Gastrointestinal Symptom Rating Scale (GSRS) (14). This scale encompasses five dimensions: abdominal distension, diarrhea, constipation, epigastric pain, and indigestion, comprising a total of 15 items. Each item is rated on a 1–7 Likert scale (1 = no discomfort, 7 = severe discomfort), yielding a total score range of 15–105.

#### 2.3.3 Assessment of nutritional status

Fasting venous blood samples (5 mL) were collected from patients in the early morning. Serum was separated by centrifugation at 3,000 rpm for 10 min and stored at −20 °C or −70 °C until analysis. Serum levels of hemoglobin (Hb), albumin (ALB), and prealbumin (PA) were measured before and after the intervention. Hb was determined using the cyanmethemoglobin method, ALB was measured using the bromocresol green method, and PA was quantified using immunoturbidimetry.

#### 2.3.4 Indicators related to neurological rehabilitation outcomes

(1) Neurological function recovery: Neurological status and recovery of consciousness before and after the intervention were assessed using the Glasgow Coma Scale (GCS) (15). The scale comprises three components: eye opening (1–4 points), verbal response (1–5 points), and motor response (1–6 points). Total scores range from 3 to 15, with higher scores indicating a better level of consciousness.

(2) Neurological injury markers: Serum samples were collected to determine the levels of glial fibrillary acidic protein (GFAP) and neuron-specific enolase (NSE) before and after the intervention in both groups. Both markers were measured using enzyme-linked immunosorbent assay.

#### 2.3.5 Patient satisfaction

Patient satisfaction regarding the therapeutic effect before and after the intervention was evaluated using the Patient Global Impression of Change (PGIC) scale (16). Total scores range from 1 (very much improved) to 7 (very much worse).

### 2.4 Statistical analysis

All data analyses were performed using SPSS 26.0 and R software. Following normality testing, continuous variables conforming to a normal distribution were expressed as mean ± standard deviation ( $\bar{x}$ ± s) and compared between groups using the independent samples *t*-test. Data not meeting the assumptions of normality were analyzed using the Mann–Whitney *U* test. Categorical variables were presented as frequencies and percentages, with between-group comparisons conducted using the chi-square test or Fisher’s exact test. Paired comparisons of pre- and post-intervention scores were analyzed using the paired *t*-test or Wilcoxon signed-rank test. PCA was performed using the FactoMineR and factoextra packages in R software. PCA was conducted as an exploratory dimensionality reduction technique to identify potential clustering patterns among outcome variables, and was not intended for confirmatory hypothesis testing. All statistical tests were two-tailed, and a *p*-value < 0.05 was considered statistically significant.

## 3 Results

### 3.1 Comparison of baseline characteristics

No significant differences were observed between the two groups regarding baseline characteristics, including age, sex distribution, body mass index (BMI), and injury type distribution (*p* > 0.05; Table 1).

**Table 1:** Comparison of baseline characteristics between groups.

Variable	Routine care group (*n* = 60)	Combined intervention group (*n* = 60)	*x^2^/t*	*P*
Age (years)	54.25 ± 11.83	53.42 ± 11.03	0.399	0.690
Sex			0.141	0.707
Male [*n* (%)]	38 (63.3%)	36 (60.0%)	–	–
Female [*n* (%)]	22 (36.7%)	24 (40.0%)	–	–
BMI (kg/m^2^)	23.19 ± 3.01	23.76 ± 2.59	−1.113	0.268
Injury type			0.153	0.985
Traffic accident [*n* (%)]	27 (45.0%)	29 (48.3%)	–	–
Fall from height [*n* (%)]	18 (30.0%)	17 (28.3%)	–	–
Fall [*n* (%)]	10 (16.7%)	9 (15.0%)	–	–
Others [*n* (%)]	5 (8.3%)	5 (8.3%)	–	–

BMI, body mass index.

### 3.2 Analysis of constipation recovery indicators

Prior to the intervention, no statistically significant differences were found between the two groups in terms of weekly frequency of completely spontaneous bowel movements, degree of defecation effort, BSS score, or total PAC-SYM score (*p* > 0.05). Following the 4-week intervention period, the combined intervention group demonstrated a significantly higher frequency of completely spontaneous bowel movements compared with the routine care group. Furthermore, the combined intervention group exhibited significantly lower scores for defecation effort and total PAC-SYM, as well as a significantly higher BSS score, relative to the routine care group. All observed differences were statistically significant (*p* < 0.05; Table 2).

**Table 2:** Analysis of constipation recovery indicators between groups.

Group	Completely spontaneous bowel movements (times/week)		Defecation effort (score)		BSS score		Total PAC-SYM score	
	Pre-intervention	Post-intervention	Pre-intervention	Post-intervention	Pre-intervention	Post-intervention	Pre-intervention	Post-intervention
Routine care (*n* = 60)	1.55 ± 0.79	2.12 ± 0.76	3.33 ± 0.54	2.52 ± 0.68	1.63 ± 0.58	2.35 ± 0.61	29.97 ± 5.40	26.38 ± 5.16
Combined intervention (*n* = 60)	1.63 ± 0.76	3.50 ± 1.03	3.35 ± 0.52	1.00 ± 0.37	1.82 ± 0.60	3.02 ± 0.62	29.22 ± 4.22	21.60 ± 5.04
*t*	−0.589	−8.349	−0.173	15.257	−1.705	−5.937	0.847	5.139
*P*	0.557	0.000	0.863	0.000	0.091	0.000	0.399	0.000

BSS, Bristol Stool Scale; PAC-SYM, Patient Assessment of Constipation Symptoms.

### 3.3 Analysis of GI function and nutritional indicators

No statistically significant between-group differences were observed at baseline regarding GSRS scores and serum levels of Hb, ALB, and PA (*p* > 0.05). Following the intervention, the combined intervention group demonstrated significantly lower GSRS scores compared with the routine care group. Conversely, serum concentrations of Hb, ALB, and PA were significantly higher in the combined intervention group than in the routine care group. All between-group differences were statistically significant (*p* < 0.05; Table 3).

**Table 3:** Comparison of GI function scores and nutritional indicators.

Group	GSRS score		Hb (g/L)		ALB (g/L)		PA (mg/L)	
	Pre-intervention	Post-intervention	Pre-intervention	Post-intervention	Pre-intervention	Post-intervention	Pre-intervention	Post-intervention
Routine care (*n* = 60)	64.40 ± 8.91	58.20 ± 8.27	113.93 ± 8.07	117.23 ± 8.10	36.67 ± 2.41	37.73 ± 2.86	180.08 ± 23.47	193.82 ± 21.69
Combined intervention (*n* = 60)	66.32 ± 9.24	49.58 ± 7.97	115.03 ± 8.79	123.25 ± 9.34	36.12 ± 2.46	40.26 ± 2.38	187.48 ± 20.06	214.59 ± 20.27
*t*	−1.156	5.812	−0.715	−3.772	1.227	−5.286	−1.858	−5.420
*P*	0.250	0.000	0.476	0.000	0.222	0.000	0.066	0.000

GI, gastrointestinal; GSRS, Gastrointestinal Symptom Rating Scale; Hb, hemoglobin; ALB, albumin; PA, prealbumin.

### 3.4 Analysis of neurological function recovery and neurological injury markers

At baseline, no statistically significant differences were detected between the two groups regarding GCS scores or serum GFAP/NSE levels (*p* > 0.05). Post-intervention, the combined intervention group exhibited significantly higher GCS scores compared with the routine care group (*p* < 0.05). However, although serum GFAP and NSE levels showed a decreasing trend in the combined intervention group, the between-group differences did not reach statistical significance (*p* > 0.05; Table 4).

**Table 4:** Comparison of GCS scores and neurological injury marker levels between groups.

Group	GCS score		GFAP (ng/L)		NSE (ng/mL)	
	Pre-intervention	Post-intervention	Pre-intervention	Post-intervention	Pre-intervention	Post-intervention
Routine care (*n* = 60)	6.20 ± 1.73	8.23 ± 2.52	6.22 ± 1.10	4.99 ± 1.02	19.10 ± 2.30	15.78 ± 2.39
Combined intervention (*n* = 60)	6.33 ± 2.20	10.67 ± 1.92	5.91 ± 1.13	4.77 ± 1.11	19.60 ± 2.69	15.30 ± 1.80
*t*	−0.370	−5.950	1.538	1.538	−1.109	1.251
*P*	0.712	0.000	0.127	0.127	0.270	0.214

GCS, Glasgow Coma Scale; GFAP, glial fibrillary acidic protein; NSE, neuron-specific enolase.

### 3.5 Patient satisfaction analysis

**Figure 1:**
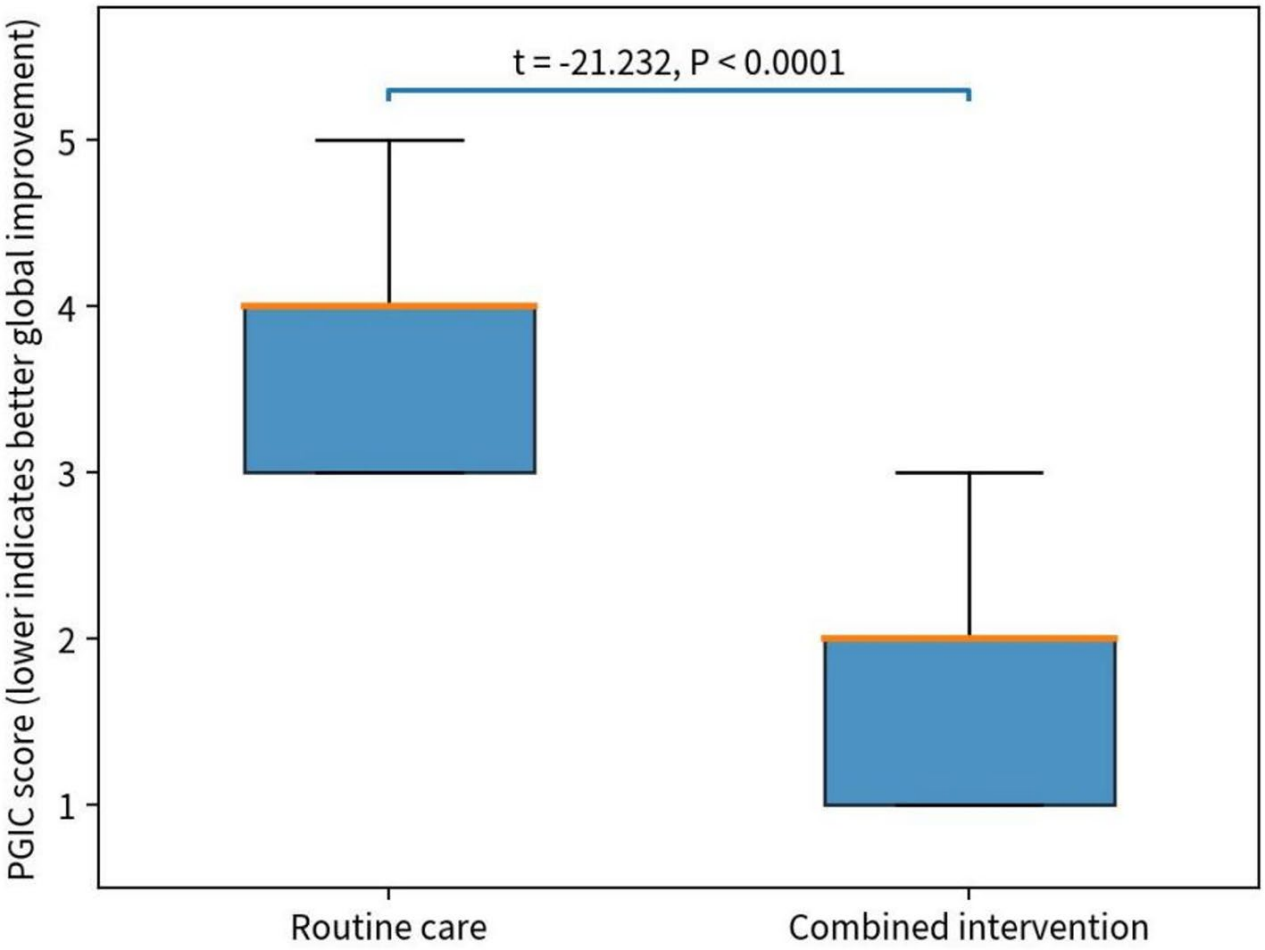
Comparison of PGIC scores between the two groups. PGIC, patient global impression of change.

Following the intervention, the PGIC score in the combined intervention group was significantly lower than that in the routine care group (1.58 ± 0.53 vs. 3.70 ± 0.56 points; *t* = −21.232, *p* < 0.05; Figure 1).

### 3.6 PCA results

To further explore potential patterns among multidimensional rehabilitation outcomes, principal component analysis (PCA) was performed on 10 post-intervention variables with significant between-group differences. These variables included frequency of completely spontaneous bowel movements, defecation effort score, BSS score, total PAC-SYM score, GSRS score, serum Hb, ALB, and PA levels, GCS score, and PGIC score.

The PCA is an unsupervised dimensionality reduction technique and was applied in this study for exploratory data visualization rather than confirmatory hypothesis testing. The first two principal components explained 48.99% of the total variance (PCA1: 38.14%; PCA2: 10.86%).

The loading structure of PCA1 suggested a general pattern reflecting co-variation among gastrointestinal function, nutritional indicators, and neurological status. PCA2 appeared to reflect a secondary pattern mainly related to the relationship between subjective symptom scores and nutritional variables. However, these interpretations are exploratory in nature and should be interpreted with caution.

**Figure 2:**
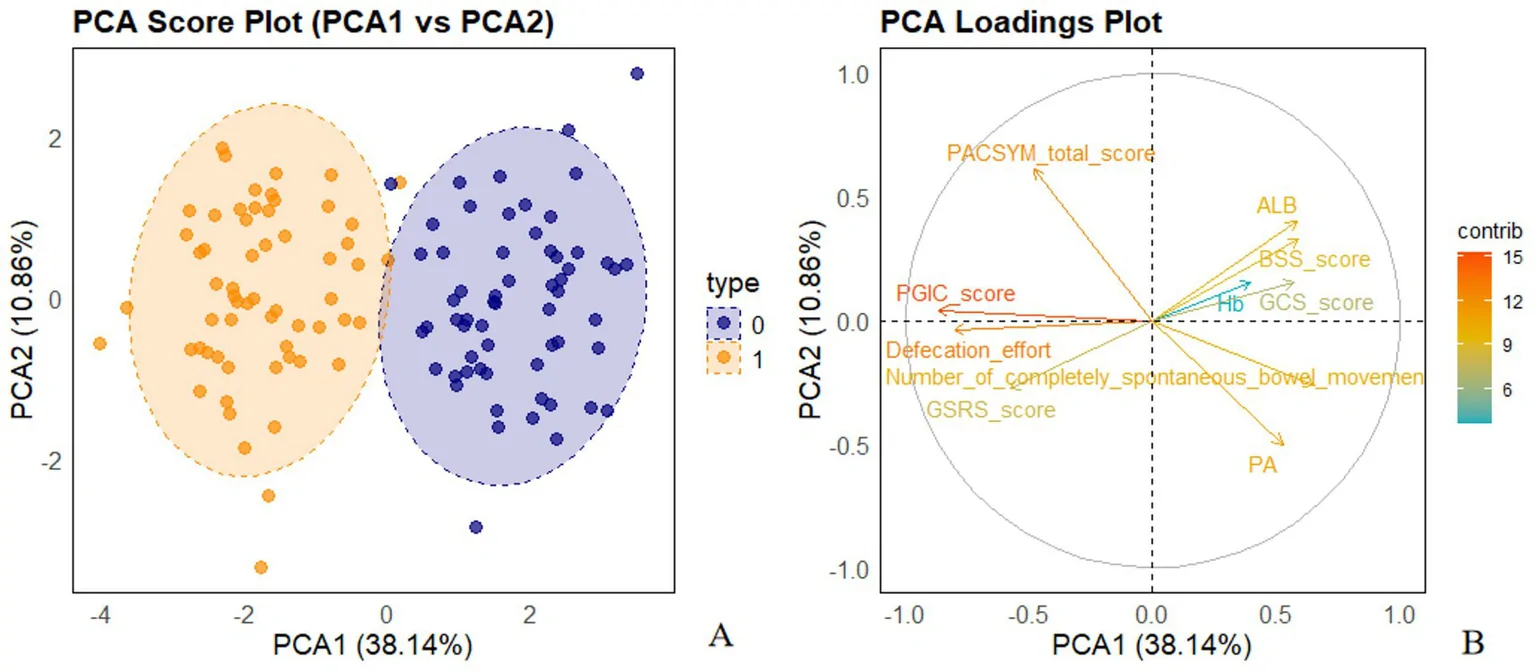
PCA of post-intervention indicators in the two groups. 1: routine care group; 0: combined intervention group. PCA, principal component analysis.

Comparison of principal component scores between groups showed higher PCA1 scores in the combined intervention group (*p* < 0.05), while no significant difference was observed for PCA2 (*p* > 0.05). These findings primarily provide a data-driven visualization of outcome structure and inter-variable relationships, rather than confirmatory evidence of treatment effects (Figure 2 and Table 5).

**Table 5:** Principal component loading matrix and variance contribution rates for post-intervention differential indicators.

Principal component	PCA1	PCA2
Number of completely spontaneous bowel movements	0.334519124	−0.250475238
Defecation effort	−0.409776031	−0.034679726
BSS score	0.301424127	0.315168312
Total PAC-SYM score	−0.245475275	0.584810383
GSRS score	−0.294416034	−0.265495049
Hb	0.202305212	0.146372003
ALB	0.29741308	0.38419719
PA	0.268838406	−0.481233699
GCS score	0.291371588	0.148353701
PGIC score	−0.442528773	0.040133093
*t*	23.529	−0.636
*P*	0.000	0.526
Variance explained percentage (%)	38.14	10.86

PCA, principal component analysis; BSS, Bristol Stool Scale; PAC-SYM, Patient Assessment of Constipation Symptoms; GSRS, Gastrointestinal Symptom Rating Scale; Hb, hemoglobin; ALB, albumin; PA, prealbumin; GCS, Glasgow Coma Scale; PGIC, Patient Global Impression of Change.

No serious adverse events were observed during the intervention period in either group. The combined intervention of Tuina and pulsed dry cupping was generally well tolerated by patients with severe traumatic brain injury.

### 3.7 Safety and tolerability analysis

Regarding tolerability, most patients were able to complete the intervention without interruption. Hemodynamic parameters remained stable throughout the treatment sessions, and no clinically significant autonomic instability (such as sudden blood pressure fluctuations, tachycardia, or oxygen desaturation) was recorded during or immediately after the procedures.

Mild and transient local skin reactions, including erythema at cupping sites, were occasionally observed in the combined intervention group. These reactions resolved spontaneously without additional treatment. No cases of skin injury, bleeding, or infection were reported. In addition, only a small proportion of patients reported mild abdominal discomfort during initial Tuina sessions, which was alleviated after adjustment of manipulation intensity. Overall, the intervention demonstrated a favorable safety profile and good clinical tolerability in this patient population.

## 4 Discussion

The rehabilitation process for patients with sTBI is inherently complex and protracted. Constipation, a prevalent complication in this population, arises from a multifactorial etiology encompassing impaired consciousness, restricted mobility, prolonged bed rest, diminished intestinal motility, pharmacotherapy, and physiological stress states (17). Beyond compromising patient comfort and amplifying nursing burden, constipation may indirectly impede holistic rehabilitation through pathways including abdominal distension, reduced appetite, and inadequate nutritional intake. While conventional management approaches, such as laxative administration, enemas, and dietary modifications, can offer transient symptomatic relief, their utility in long-term management is often constrained by issues of tolerance, adverse effects, and patient dependence. Consequently, the exploration of safe, reproducible, non-pharmacological interventions amenable to integration into routine nursing workflows holds substantial pragmatic significance.

Constipation in patients with severe traumatic brain injury is a multifactorial condition influenced by several interacting mechanisms. In addition to impaired central autonomic regulation following brain injury, prolonged bed rest, reduced physical activity, and the frequent use of sedative or opioid medications in neurocritical care settings contribute to decreased gastrointestinal motility. Nutritional restrictions and altered enteral feeding tolerance may further exacerbate bowel dysfunction. From a neurogastroenterological perspective, disruption of the brain–gut axis has been recognized as an important mechanism underlying gastrointestinal dysregulation in critically ill neurological patients. Previous studies have suggested that impaired neuroautonomic signaling and systemic inflammatory responses may affect intestinal motility and bowel function (18, 19).

This study evaluated the clinical applicability of Tuina combined with pulsed dry cupping in constipation management and associated functional recovery in patients with sTBI, adopting a multidimensional framework encompassing constipation symptoms, GI function, nutritional status, neurological function-related indicators, and patient satisfaction.

### 4.1 Association between Tuina combined with pulsed dry cupping and constipation-related outcomes

The findings of this study demonstrated that, following the intervention, the combined intervention group exhibited a significant increase in the frequency of completely spontaneous bowel movements, a reduction in defecation effort, an improvement in BSS scores, and a decrease in total PAC-SYM scores. These results indicate a substantial association between the combined Tuina and pulsed dry cupping protocol and the amelioration of constipation symptoms. Tuina, a widely utilized traditional Chinese medicine rehabilitation nursing technique, applies manual stimulation to the abdomen and associated regions. This approach may contribute to the promotion of intestinal peristalsis, improvement of colonic transit, and facilitation of the defecation reflex, thereby alleviating defecatory difficulty (20).

Pulsed dry cupping, characterized by its rhythmic negative pressure stimulation and localized mechanical traction, may exert therapeutic effects through multiple mechanisms. These potentially include the reduction of abdominal soft tissue tension, enhancement of regional blood circulation and fluid return, and modulation of autonomic nervous system regulation. Such effects may collectively mitigate abdominal distension and support the restoration of defecatory propulsion. Relative to singular interventions, the combined application of Tuina and pulsed dry cupping may produce complementary therapeutic actions: Tuina primarily emphasizes promotion of motility and clearance of stasis, whereas cupping focuses on stimulation and regulation. This synergy may contribute to more stable and pronounced symptomatic improvement (21). Nevertheless, it is imperative to emphasize that the present study, employing a controlled design, primarily elucidates statistical associations and differences between the intervention and outcome improvements. Elucidation of the specific physiological mechanisms necessitates further investigation incorporating more refined physiological parameters or dedicated mechanistic studies.

### 4.2 Clinical significance of Tuina combined with pulsed dry cupping in improving GI function and nutritional status

In this study, the combined intervention group demonstrated significantly lower GSRS scores and significantly higher serum levels of Hb, ALB, and PA compared with the routine care group. These findings suggest that, beyond alleviating constipation, this combined intervention may be associated with the restoration of GI function and enhancement of nutritional status. For patients with sTBI, GI dysfunction frequently manifests as delayed gastric emptying, abdominal distension, diminished appetite, or poor enteral feeding tolerance, which can subsequently impede the implementation of EN and impair protein synthesis (22, 23). Relief from constipation and associated GI discomfort may improve conditions for nutrient intake and absorption, potentially leading to favorable trends in nutritional biochemical markers.

From a clinical nursing perspective, Tuina and pulsed dry cupping, as non-pharmacological interventions, offer advantages of reproducible administration and relative ease of integration into standard nursing workflows. These modalities may thus provide a supplementary approach for the long-term, sustained management of constipation and GI dysfunction (24). However, it is important to acknowledge that nutritional indicators may be influenced by a multitude of factors beyond the intervention itself, including underlying disease severity, enteral feeding strategies, infectious status, and metabolic stress responses. Subsequent research should endeavor to more rigorously control for potential confounding variables and assess the sustainability of nutritional improvements and their association with clinical outcomes over extended follow-up periods.

### 4.3 Preliminary observations regarding neurological function-related outcomes

Emerging concepts surrounding the gut-brain axis suggest potential reciprocal interactions between GI function, nutritional status, and neuroinflammatory responses as well as neurological recovery processes (25). The present study found higher GCS scores in the combined intervention group compared with the routine care group. However, given the observational design, these findings should be interpreted as associations rather than evidence of a causal effect on neurological recovery. The underlying mechanisms remain speculative. Although Tuina and pulsed dry cupping may theoretically influence gastrointestinal function through mechanical stimulation and potential modulation of autonomic nervous system activity, no direct physiological or autonomic measurements were conducted in this study. Therefore, these interpretations should be considered hypothesis-generating rather than confirmatory.

Although serum GFAP and NSE levels showed a decreasing trend in the combined intervention group, the between-group differences were not statistically significant. Therefore, no definitive conclusions can be drawn regarding the effect of the intervention on neurological injury biomarkers. These results may be influenced by factors such as baseline injury severity, temporal dynamics of brain injury, and the limited observation period. Overall, the interpretation of neurological outcomes should remain cautious, and further studies with larger sample sizes and longer follow-up are required to validate these findings.

### 4.4 Subjective patient-reported benefit and multidimensional rehabilitation outcome evaluation

Patient satisfaction serves as an important indicator reflecting the acceptability, comfort, and overall subjective perception of therapeutic benefit associated with an intervention. In the present study, PGIC scores were significantly lower in the combined intervention group compared with the routine care group, indicating a substantial advantage of the combined intervention in terms of patients’ global impression of improvement. This favorable outcome may be attributable to a constellation of factors, including alleviation of constipation symptoms, relief from abdominal distension and discomfort, enhanced GI tolerance, and improved comfort during nursing care procedures.

Furthermore, the PCA results showed different distribution patterns of multidimensional indicators related to constipation symptoms, gastrointestinal function, and recovery-related variables between the two groups. This observation reflects potential clustering characteristics of outcome variables at a multivariate level. However, this pattern description should not be interpreted as evidence of a synergistic therapeutic effect. It is important to note that PCA is an exploratory statistical method used for dimensionality reduction and data visualization. Therefore, its results are primarily intended to describe variable structure and inter-variable relationships rather than to support causal or confirmatory conclusions. The main evidence for treatment effects should be based on primary between-group comparisons of clinical outcomes.

A further important consideration is the difficulty in isolating the specific effects of individual components within the combined intervention. In the present study, Tuina, pulsed dry cupping, and standard nursing care were applied in an integrated manner as part of routine clinical practice. Therefore, it is not possible to determine the independent contribution of each component to the observed outcomes. The findings should thus be interpreted as reflecting the overall effect of a combined rehabilitation nursing strategy rather than the isolated efficacy of any single intervention. Future studies with factorial or dismantling designs are needed to further clarify the relative contribution of each component.

### 4.5 Limitations and future research directions

Several limitations inherent to this study warrant acknowledgment. First, this investigation was conducted as a single-center controlled study with a relatively modest sample size. Consequently, generalizability of the findings should be approached cautiously. Second, the intervention and observation period was limited to 4 weeks, precluding assessment of long-term maintenance effects and extended functional outcomes. Third, despite the inclusion of multidimensional indicators, the potential influence of residual confounding factors, including injury severity, nutritional support strategies, concurrent medication use, and complications, cannot be entirely excluded. Future research endeavors should prioritize the conduct of multicenter studies to facilitate the standardization of intervention protocols and operational procedures. Extended follow-up durations are essential to evaluate durability of effects and long-term functional prognoses. Incorporation of more objective measures, such as colonic motility parameters, definitive nutritional endpoints, and validated neurological function outcome scales, will be instrumental in enhancing the quality of evidence and bolstering the clinical generalizability of these findings. Given the prospective observational design of this study, the observed associations should be interpreted cautiously, and causal effects cannot be inferred. Several clinically important factors that may influence gastrointestinal and neurological recovery, including injury severity, sedation and analgesia exposure, enteral nutrition strategies, bowel regimens, mobilization status, and neurosurgical interventions, were not fully captured in this study, which may introduce potential residual confounding.

## 5 Conclusion

Tuina combined with pulsed dry cupping is significantly associated with improved constipation symptoms, enhanced GI function recovery, better nutritional status, and increased patient satisfaction in patients with sTBI. As a beneficial supplement to conventional nursing care, this combined intervention demonstrates satisfactory feasibility and notable clinical application value. However, its effects on indicators related to neurological function recovery require further validation through investigations involving larger sample sizes, extended longitudinal follow-up, and more rigorous study designs.

## Data Availability

The original contributions presented in the study are included in the article/supplementary material, further inquiries can be directed to the corresponding author.
